# Prospective, Population‐Based Evaluation of Costs and Outcomes of Status Epilepticus According to the New Definition

**DOI:** 10.1111/ene.70777

**Published:** 2026-10-20

**Authors:** Clara Jünemann, Meike Menche, Jens‐Peter Reese, Felix Rosenow, Martin Jünemann, Christoph Best, Michael Teepker, Christian Roth, Ingrid Sünkeler, Elisabeth Pryss, Leona Möller, Katja Menzler, Lars Timmermann, Susanne Knake, Adam Strzelczyk

**Affiliations:** ^1^ Epilepsy Center Hessen Philipps University Marburg Germany; ^2^ Center for Epidemiology and Biometry Julius Maximilians University Würzburg Germany; ^3^ Public Health and Epidemiology, Health Services Research, and Health Data Science Technische Hochschule Mittelhessen Giessen Germany; ^4^ Epilepsy Center Frankfurt Rhine‐Main Goethe University Frankfurt am Main Germany; ^5^ Clinic for Neurology Justus Liebig University Gießen Germany; ^6^ Vitos Clinic for Neurology Weilmünster Germany; ^7^ Hardtwald Clinic I for Neurology Bad Zwesten Germany; ^8^ Department of Neurology Klinikum Kassel Kassel Germany; ^9^ DRK Kliniken Nordhessen Kassel Germany; ^10^ BDH Clinic for Neurology Braunfels Germany; ^11^ Clinic for Internal Medicine Frankenberg Germany; ^12^ Section Epileptology, Department of Neurology Jena University Hospital Jena Germany

**Keywords:** costs, economic burden, outcome, seizure, status epilepticus

## Abstract

**Background:**

To address the paucity of data on outcomes and costs of status epilepticus (SE) following the updated 2015 International League Against Epilepsy (ILAE) definition and changes in clinical practice guidelines in Germany.

**Methods:**

This prospective population‐based study (October 2018–March 2020) included adult patients from postal code area prefix 35, representative of Germany, diagnosed with SE according to the 2015 ILAE definition. Costs were calculated using German Diagnosis‐Related Groups (G‐DRG).

**Results:**

In total, 181 patients were included. Mean inpatient costs were €10,543 (median €6376; range €845–€133,267), corresponding to €939 per hospital day (median €580.8; range €76.8–€12,115). Mean length of stay (LOS) was 11 days (median 9; range 1–67). In univariable analyses, super‐refractory SE was associated with higher costs and longer LOS (both *p* < 0.001), while acute symptomatic etiology was associated with longer LOS (*p* = 0.006). On multivariable analysis, mechanical ventilation was the strongest independent predictor of costs (Exp(B) = 2.918, 95% CI 1.753–4.857; *p* < 0.001), and etiology was overall significantly associated with cost variation (*p* = 0.022), with acute symptomatic SE showing the highest adjusted costs. Discharge destination correlated with SE severity, with most patients with non‐refractory SE discharged home (*p* = 0.028).

**Conclusions:**

This study provides population‐based data on costs and outcomes of SE in Germany, serving as a basis for future cost‐effectiveness analyses. Compared with earlier studies, lower direct costs and shorter LOS were observed. Identification of high‐risk constellations may support clinical decision‐making and optimize monitoring, treatment escalation, and allocation of intensive care resources.

## Graphical abstract

This prospective population‐based study analyzed inpatient costs and outcomes of status epilepticus (SE) in 181 adult patients in Germany. Mean direct inpatient costs were €10,543 per admission, with super‐refractory SE and acute symptomatic etiology associated with the highest costs and longest length of stay; most patients with non‐refractory SE were discharged home. On multivariable analysis, mechanical ventilation was the strongest independent predictor of inpatient costs, highlighting the dominant role of intensive care resource utilization in SE.

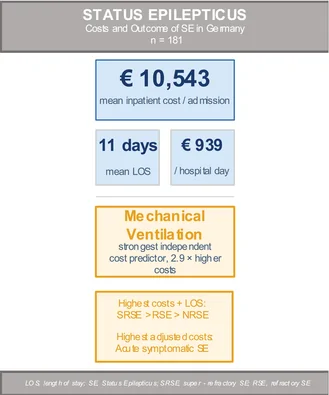

## 1 Introduction

Status epilepticus (SE) is one of the most common life‐threatening emergencies in neurology and is associated with significant mortality and morbidity [1]. Before the 2015 updated International League Against Epilepsy (ILAE) definition, the consensus definition of SE was an epileptic seizure lasting more than 30 min or a series of seizures without full recovery of consciousness that requires the initiation of intensive medical therapy [2]. A population‐based study investigated the incidence of SE in an area in the central part of Germany (postal code area prefix 35) from 1998 to 2000, reporting an incidence of 17.1 in 100,000 and a mortality rate of 9.3% [1].

However, experimental and empirical data suggest that irreversible damage to neurons and a progressively worse response to pharmacological interventions occur after only a few minutes of continuous seizure activity [2, 3]. Current data from prior cohort studies suggest that patients have a significantly worse outcome with increased SE duration. Indeed, data already show a difference in outcome between treatment of less than and more than 30 min [4]. Earlier literature had already suggested an operational definition of 5 min [5]. It is now internationally accepted and recommended by a task force of the International League Against Epilepsy (ILAE) that consistent treatment of generalized convulsive SE should be administered after 5 min of clinical seizure activity [2].

While a multicentre cohort study in Germany reported comparable inpatient costs with the new definition of SE to those recorded with the previous definition, this study did not consider the consequent change in incidence [6], which would be expected to change costs in addition to demographic shifts. The change in definition would also be expected to alter outcomes, such as functional outcomes, affective measures (mood, emotional experience), and even mortality. Therefore, this study aimed to examine current health economic and epidemiological data after the introduction of the new definition of SE, as previous studies have not considered this revised definition.

## 2 Patients and Methods

### 2.1 Study Design

The data for the present study were collected in the same geographical region (identical postal code area) and followed the same study design and analytical approach as the earlier 1999 investigation on the incidence of status epilepticus in adults in Germany [1]. It included all adult patients who lived in the postal code area prefix 35, which encompasses the primary care area of Marburg and Giessen University Hospital and attended one of the 14 participating neurological hospitals in the study area (or an adjacent area) between 1 October 2018 and 1 March 2020. All patients with a diagnosis of status epilepticus were initially identified, and the diagnosis was subsequently confirmed by the study team through detailed review of the medical records. For this purpose, the hospital database of Marburg University Hospital was retrospectively screened for discharge diagnoses of status epilepticus using the German modification of the International Classification of Diseases, 10th Revision (ICD‐10‐GM), code G41. To maximize case ascertainment, all participating hospitals were contacted weekly by telephone and fax regarding potential SE cases. Suspected cases were reviewed on site within 3 days. In addition, retrospective database screening was performed at the two largest participating university hospitals (Marburg and Giessen) to identify potentially missed cases. Individual chart review was used to verify eligibility and avoid duplicate inclusion of transferred patients.

The epidemiological characteristics of this population‐based cohort, including age, sex, etiology, and case detection strategy, have been described previously. This study is based on the same cohort but reports, for the first time, health economic outcomes, inpatient costs, and cost‐related clinical factors [7]. This study assessed the completeness of case identification within the study region, assuming no relevant underreporting. Seizure types and SE were categorized according to the current ILAE classification frameworks for SE and seizure types. Consistent with the German guideline, patients were considered to have SE if clinical presentation and/or EEG findings indicated seizure activity lasting longer than 5 min, independent of the specific SE subtype [8, 9, 10]. Patients who had post‐hypoxic brain damage with myoclonus, patients whose seizures lasted less than 5 min, patients < 18 years, and patients residing outside the postal code area prefix 35 were excluded.

The study area was chosen because it has a population structure that is considered representative of Germany. According to epidemiological data from the German Federal Statistical Office for 2019, the study area comprised 981,068 adults, of whom 51% were female, and showed a slight tendency towards younger age groups than the national average of 329,430 individuals aged over 60 years [11]. It also examined the primary care area of the university hospital Marburg in more detail, as this area is also considered representative of Germany due to its population distribution [12].

To collect the data, we designed a questionnaire containing questions on health economic aspects of SE, such as costs incurred by patients with SE, as well as questions on their health status and life circumstances before and after experiencing SE. After 30 days, the patients or their attendants were recontacted to inquire about their current health status, any potential further hospitalisations, and to assess their quality of life. The data were collected, saved, and analyzed anonymously by the Marburg study team.

To facilitate a direct comparison, the same subtypes of SE were used as in the 2016 study, consistent with ILAE recommendations [13]. For those with acute symptomatic SE, the triggering event (e.g., an ischemic stroke, intracerebral hemorrhage, or meningitis) was identified 1–7 days previously [14]. In addition, a group of seizures with a remote etiology was defined when there was already known brain damage and a history of epilepsy. New‐onset remote SE was defined as a seizure event that occurred for the first time in a patient with a known brain injury. Finally, other seizures were combined where none of these groups applied. For patients without sufficient information, such as those who left the clinic prematurely, the seizures were classified as unknown [13, 15].

Missing data were classified as ‘unknown’, the analyses were carried out based on the available cases. The missing values were primarily due to early discharges, incomplete emergency documentation and the unavailability of patients at the follow‐up examination, which was mainly attributable to their state of health or transfers to care homes or rehabilitation clinics and the resulting lack of contact.

The study was performed in accordance with the STROBE guidelines for observational studies in epidemiology and received ethical approval from the Ethics Committee of Philipps University of Marburg (reference number: AZ 42/18). Written informed consent was obtained from all patients or their legal representatives prior to study participation in accordance with the Declaration of Helsinki. The corresponding epidemiological data were published in a previous paper [7].

### 2.2 Instruments

The Epidemiology‐based Mortality Score in Status Epilepticus (EMSE) [16], the Status Epilepticus Severity Score (STESS) and the Charlson Comorbidity Index (CCI) were used to assess morbidity and outcome predictors [17, 18]. Independence was assessed before the index admission and 30 days after discharge using the Barthel Index and the modified Rankin Scale (mRS) to evaluate outcomes [19]. Finally, the Neurological Disorders Depression Inventory for Epilepsy (NDDI‐E) and the EuroQol five‐dimension, five‐level (EQ‐5D‐5L) questionnaire were used to assess patients' physical and mental health status and their overall quality of life [20, 21].

### 2.3 Cost Assessment

All costs were submitted to the Marburg study team by each participating hospital and calculated using the German Diagnosis‐Related Groups (G‐DRG) according to the German guidelines for conducting economic evaluations in healthcare [22], taking into account possible ventilation, duration of ventilation, form of ventilation, and duration of intensive care treatment. Costs were estimated using the German Diagnosis‐Related Groups (G‐DRG) system by multiplying the individual DRG cost weight by the applicable base rate. The cost analysis therefore reflects the DRG‐based reimbursement amounts for the reference stay from the perspective of the statutory health insurer but does not represent a micro‐costing analysis. The duration of SE was determined based on medical documentation, EEG, and information provided by the emergency physician or emergency medical services.

The billings were also queried to identify all patients with SE as the primary or secondary diagnosis using the G‐DRG. The respective hospital provided information about the onset, treatment, and course of SE, preexisting epilepsy syndrome, etiology of SE, concomitant diseases, current anti‐seizure medications (ASMs), and other medications, discharge destination, and outcome. The length of inpatient stay (LOS) and inpatient treatment costs were used as variables in determining factors driving costs. Clinical variables were tested as potential cost drivers based on previous studies, using univariate analyses.

### 2.4 Statistical Analysis

The German Federal Statistical Office was consulted to obtain the number of adults registered in the postal code area prefix 35 [11]. The likelihood of experiencing SE as a function of sex and age was examined using chi‐square tests. Then, the incidence was calculated by dividing the number of patients experiencing their first SE episodes in the study area by the number of adults residing in the study area. The Clopper–Pearson confidence interval (CI) was used to calculate the one‐year incidence rate in the study area, adjusting for age and sex. Population‐based conclusions were then drawn based on the incidence and the calculated cost per patient. Finally, the incidence was used to determine the costs and outcomes following the change in the definition of SE. A possible correlation between costs and outcomes was investigated using various parameters, such as etiology, course, and demographic characteristics.

The statistical analyses were performed using IBM SPSS Statistics (version 29). Costs, LOS, and duration of ventilation were calculated per inpatient stay for all SE‐related treatments. The variables are summarized as the mean ± standard deviation (SD), median (range: minimum–maximum), or percentage (number). In addition, 95% CIs are reported, calculated using the bootstrap method according to the bias‐corrected accelerated approach. Categorical variables were compared using the chi‐square test or Fisher's exact test, as appropriate; continuous variables were analyzed using the *t*‐test or nonparametric tests, depending on data distribution. For skewed distributions, such as cost data, the nonparametric Mann–Whitney *U* test was applied. To assess the robustness of the results, a sensitivity analysis was performed by excluding outliers (cost values exceeding the mean + 3 SD; *n* = 5) and applying parametric tests (independent *t*‐test, ANOVA) to the remaining cases. A *p*‐value of ≤ 0.05 was considered statistically significant. To identify independent predictors of inpatient costs, a multivariable generalized linear model (GLM) with a Gamma distribution and log link function was applied, as cost data were right‐skewed. The model included SE severity (non‐refractory SE (NRSE) vs. refractory SE (RSE)/super‐refractory SE (SRSE)), etiology, mechanical ventilation, age, and pre‐admission mRS as covariates. Exponentiated coefficients (Exp(*B*)) with 95% Wald confidence intervals were reported as cost multipliers. The analysis was restricted to cases with complete data on all model variables.

Missing data were categorized as “unknown”, and analyses were performed based on available cases.

## 3 Results

### 3.1 The Characteristics of Patients With SE

This study examined 181 adult patients who lived in the postal code area prefix 35 and experienced SE. Table 1 shows sociodemographic and clinical characteristics by SE etiology. The mean age of all patients was 66.0 ± 18.7 years (median = 69.5, range = 20–94), with most aged 60 years or older (*n* = 114, 63%). Eighty‐seven were male (48%), 78 were female (43%), and sex was unavailable in 16 patients (9%). Most patients presented with generalized and convulsive SE (57.5%), followed by focal SE (24.8%); 11.6% were NCSE (non‐convulsive SE), 1.7% were absences, and 4.4% were unknown.

**TABLE 1:** Sociodemographic and clinical characteristics by SE etiology.

Variable	Category	All patients (*n* = 181)	New‐onset, remote (*n* = 23)	Acute symptomatic (*n* = 48)	Remote (*n* = 75)	Other (*n* = 35)
Age (years)	mean ± SD	66 ± 18.7	73 ± 17	69 ± 15	60 ± 21	73 ± 11
	range	20–94	29–94	39–93	20–93	49–90
Sex, % (*n*)	male	48.1 (87)	60.9 (14)	52.1 (25)	53.3 (40)	22.9 (8)
	female	43.1 (78)	39.1 (9)	45.8 (22)	46.7 (35)	34.3 (12)
	unknown	8.8 (16)	0 (0)	2.1 (1)	0 (0)	42.9 (15)
Entity, % (*n*)	cerebrovascular	34.8 (63)	69.6 (16)	39.6 (19)	34.7 (26)	5.7 (2)
	degenerative	2.8 (5)	4.3 (1)	0 (0)	1.3 (1)	8.6 (3)
	infectious	5.5 (10)	0 (0)	10.4 (5)	6.7 (5)	0 (0)
	tumor	10.5 (19)	8.7 (2)	14.6 (7)	12 (9)	2.9 (1)
	traumatic	1.7 (3)	0 (0)	2.1 (1)	2.7 (2)	0 (0)
	other	18.8 (34)	17.4 (4)	25 (12)	24 (18)	0 (0)
	unknown	26 (47)	0 (0)	8.3 (4)	18.7 (14)	82.9 (29)
mRS before	0–3	54.1 (98)	69.6 (16)	66.7 (32)	50.7 (38)	34.2 (12)
admission, % (*n*)	4–5	33.7 (61)	30.4 (7)	29.2 (14)	46.7 (35)	14.2 (5)
	unknown	12.2 (22)	0 (0)	4.2 (2)	2.7 (2)	51.4 (18)
CCI, % (*n*)	0–4	71.8 (130)	69.6 (16)	77.1 (37)	86.7 (65)	34.3 (12)
	≥ 5	13.3 (24)	21.7 (5)	16.7 (8)	9.3 (7)	11.4 (4)
	unknown	14.9 (27)	8.7 (2)	6.3 (3)	4 (3)	54.3 (19)
STESS, % (*n*)	0–3	55.2 (100)	73.9 (17)	45.8 (22)	76 (57)	11.4 (4)
	4–6	32.0 (58)	21.7 (5)	47.9 (23)	22.7 (17)	37.1 (13)
	unknown	12.7 (23)	4.3 (1)	6.3 (3)	1.3 (1)	51.4 (18)
Acute treatment with benzodiazepines	No	30.9 (56)	13.0 (3)	33.3 (16)	21.3 (16)	60.0 (21)
before admission, % (*n*)	Yes	68.5 (124)	82.6 (19)	66.7 (32)	78.7 (59)	40.0 (14)
	unknown	0.6 (1)	4.3 (1)	0 (0)	0 (0)	0 (0)
Severity, % (*n*)	NRSE	22.7 (41)	21.7 (5)	14.6 (7)	30.7 (23)	17.1 (6)
	RSE	24.3 (44)	17.4 (4)	25.0 (12)	25.3 (19)	25.7 (9)
	SRSE	24.3 (44)	34.8 (8)	41.7 (20)	16.0 (12)	11.4 (4)
	unknown	28.7 (52)	26.1 (6)	18.8 (9)	28 (21)	45.7 (16)
mRS at	0–3	35.4 (64)	43.5 (10)	31.3 (15)	42.7 (32)	20 (7)
discharge, % (*n*)	4–6	41.4 (75)	52.2 (12)	64.6 (31)	53.3 (40)	28.6 (10)
	unknown	23.2 (42)	4.3 (1)	4.2 (2)	4.0 (3)	51.4 (18)

Abbreviations: CCI, Charlson Comorbidity Index; mRS, modified Rankin Scale; NRSE, non‐refractory status epilepticus; RSE, refractory status epilepticus; SD, standard deviation; SE, status epilepticus; SRSE, super‐refractory status epilepticus; STESS, Status Epilepticus Severity Score.

Patients with new‐onset remote SE were older than those with acute symptomatic or remote SE (Table 1). 27% had acute symptomatic SE, 41% had remote SE, and 13% had new‐onset remote SE. Regarding causes, 35% had an identified cerebrovascular cause, 10% were related to tumors, 5% were related to infections, 3% were related to degenerative disorders, and 2% were related to traumatic brain injuries, while 19% were related to other causes.

Sex distribution differed only slightly between acute symptomatic SE and remote SE but biased toward males for new‐onset remote SE (61% vs. 39%). The patients with remote SE exhibited a higher degree of disability (mRS > 3) at admission (47%) than those with new‐onset remote SE (30%) or acute symptomatic SE (29%). In contrast, the patients with acute symptomatic SE exhibited a greater degree of disability at discharge (67%) than those with new‐onset remote SE (52%) and remote SE (53%). Moreover, the proportion of patients with a STESS of ≤ 3 was greater among those with new‐onset remote SE (78%) and remote SE (79%) than among those with acute symptomatic SE (48%), indicating less severe SE.

The five most common comorbidities were cerebrovascular diseases, diabetes mellitus without end‐organ damage, dementia, cancers, and renal insufficiency. Among those with acute symptomatic SE, SRSE was the most common.

The mean duration was 509.8 min 1611.4 (median = 30, range = 5–7200), and approximately half of the recorded durations (51%) were between 5 and 30 min.

### 3.2 Diagnosis and Treatment

NRSE was recorded for 23% of patients, while RSE and SRSE were reported for 24% of patients. We considered the mRS to capture the outcome after SE. Compared to at admission, fewer patients had an mRS of 0–3 at discharge, whereas more patients had an mRS of ≥ 4 (Figure 1).

**FIGURE 1:**
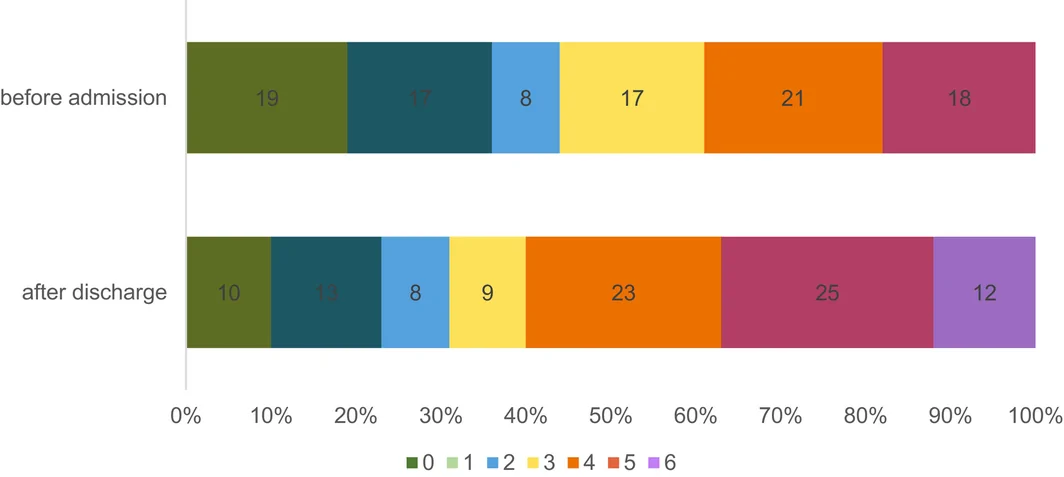
Patients' mRS scores before admission and after discharge. SE, status epilepticus.

Table 2 shows patients' sociodemographic and clinical characteristics by SE severity. Statistical significance was only observed for destination at discharge. Patients with RSE/SRSE tended to have a higher proportion of mRS 4–6 at discharge than those with NRSE. A significant correlation existed between SE severity and the discharge destination (*n* = 114), as more than 70% of the patients with NRSE (*n* = 35) were discharged home (*p* = 0.028).

**TABLE 2:** Patients' sociodemographic and clinical characteristics by SE severity.

Variable	Category	RSE/SRSE	NRSE	*p*
Age (years), mean ± SD		65.01 ± 17.8	65.35 ± 18.56	0.924
Sex, % (*n*)	male	54.8 (46)	52.6 (20)	0.327
	female	45.2 (38)	44.7 (17)	
	other	0 (0)	2.6 (1)	
Entity, % (*n*)	cerebrovascular	44.3 (31)	58.6 (17)	0.306
	degenerative	2.9 (2)	3.4 (1)	
	infectious	10 (7)	3.4 (1)	
	tumor	11.4 (8)	13.8 (4)	
	traumatic	0 (0)	3.4 (1)	
	other	31.4 (22)	17.2 (5)	
mRS before	0–3	68.4 (54)	61.1 (22)	0.447
admission, % (*n*)	4–5	31.6 (25)	38.9 (14)	
CCI, % (*n*)	0–4	84.2 (64)	88.9 (32)	0.509
	≥ 5	15.8 (12)	11.1 (4)	
Acute treatment with	no	25.3 (22)	39 (16)	0.112
benzodiazepines, % (*n*)	yes	74.7 (65)	61 (25)	
STESS, % (*n*)	0–3	64.1 (50)	69.4 (25)	0.576
	4–6	35.9 (28)	30.6 (11)	
mRS at	0–3	43.7 (31)	54.5 (18)	0.301
discharge, % (*n*)	4–6	56.3 (40)	45.5 (15)	
Destination on	death	10.1 (8)	2.9 (1)	**0.028**
discharge, % (*n*)	home	41.8 (33)	71.4 (25)	
	nursing home	8.9 (7)	11.4 (4)	
	rehabilitation	27.8 (22)	8.6 (3)	
	other hospital	11.4 (9)	5.7 (2)	

*Note:* Unreported severity, *n* = 52. Number of patients with missing data (‘unknown’) among those with known severity (*n* = 129), per variable: sex 7, entity 30, mRS before admission 14, CCI 17, acute treatment with benzodiazepines 1, STESS 15, mRS at discharge 25, destination on discharge 15.

Abbreviations: CCI, Charlson Comorbidity Index; mRS, modified Rankin Scale; NRSE, non‐refractory SE; RSE, refractory SE; SE, status epilepticus; SRSE, super‐refractory SE; STESS, Status Epilepticus Severity Score.

Regarding treatment, benzodiazepines were the most administered first‐line therapy (69%). Midazolam (< 10 mg) was administered to 43% of the patients, and lorazepam (< 4 mg) was administered to 27%. Levetiracetam was the most common ASM administered as first‐line therapy (14%). Imaging was performed in 76% of patients, with computed tomography the most common initial modality (99% of all initial images), and magnetic resonance imaging performed during hospital stay for 45% of the patients. An electroencephalogram was performed on 83% of the patients.

### 3.3 Inpatient Treatment Costs and Factors Driving Costs

Overall, the mean cost was €10,543 per patient per admission (median = €6376, range = €845–€133,267), and the mean LOS was 11 days (median = 9, range = 1–67), resulting in a mean cost of €939 per treatment day (median = €580.8, range = €76.8–€12,115.2).

Acute symptomatic SE caused the highest costs (mean = €20,012.0, median = €11,262.6, *p* = 0.053), followed by new‐onset remote SE (mean = €12,137.55, median = €6344.7) and remote SE (mean = €8879.0, median = €3785.5). In addition, SRSE also resulted in the highest costs (mean = €32,064.4, median = € 26,722.7; Figure 2A), and the longest LOS (mean = 21.4 days, median = 9; Figure 2B), followed by RSE (mean = €6469.7, median = € 4425; mean = 11.5 days, median = 12.5 days) and NRSE (mean = €6157.5, median = € 2769.6; mean = 10.9 days, median = 10 days).

**FIGURE 2A:**
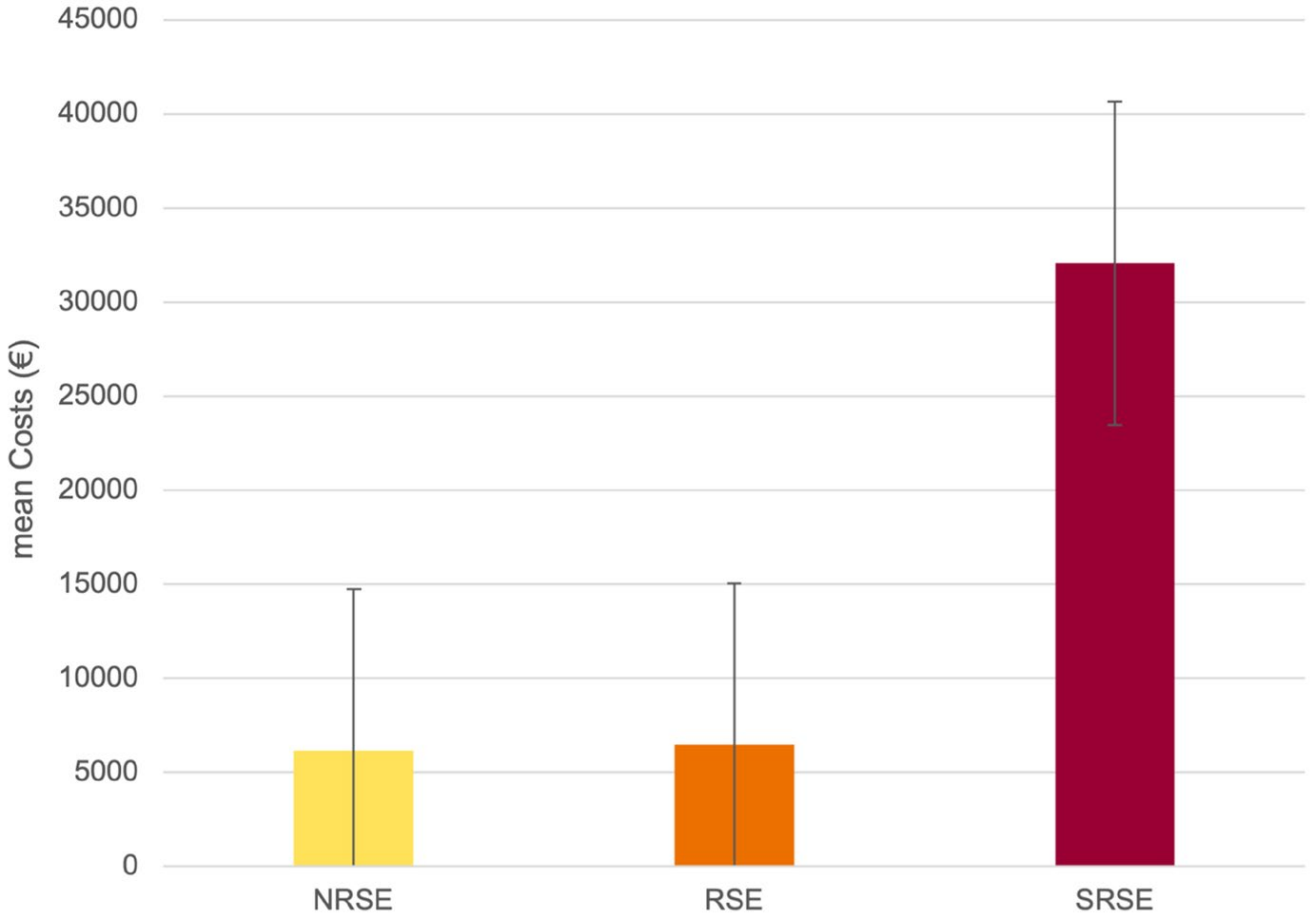
Mean costs and standard error by SE course. NRSE, non‐refractory status epilepticus; RSE, refractory status epilepticus; SRSE, super‐refractory status epilepticus.

**FIGURE 2B:**
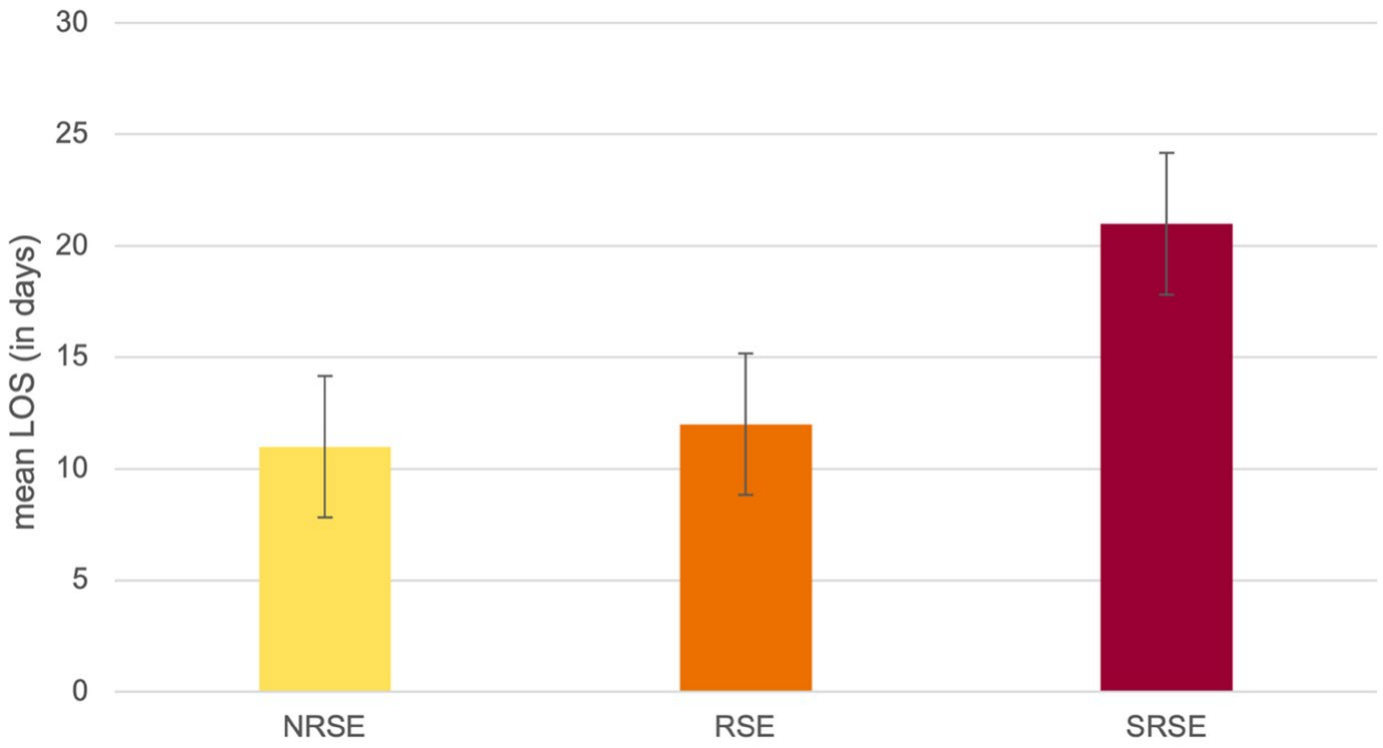
Mean LOS and standard error by SE course. NRSE, non‐refractory status epilepticus; RSE, refractory status epilepticus; SRSE, super‐refractory status epilepticus.

As shown in Table 3 SRSE/RSE (*p* < 0.001) was associated with significantly higher costs. In addition, acute symptomatic SE (*p* = 0.006) and SRSE (*p* < 0.001) were associated with significantly longer LOS. A sensitivity analysis excluding cost outliers (*n* = 5) using parametric tests confirmed these findings. SE severity remained significantly associated with costs (*p* = 0.004) and etiology showed a similar pattern, with acute symptomatic SE associated with the highest costs (*p* = 0.017).

**TABLE 3:** Direct costs (in €) and LOS (in days).

	n	mean	SD	95.0% CI	*p*
Direct costs					
Etiology					
New‐onset, remote	19	12,137.55	18,489.18	[6099, 20,340]	0.053
Acute symptomatic	45	20,012.26	22,519.91	[14,054, 27,076]	
Remote	63	8879.30	17,846.82	[5572, 13,155]	
other	15	14,518.65	26,097.02	[5589, 25,641]	
Severity					
RSE	32	6469.69	5658.06	[4723, 8327]	**< 0.001**
SRSE	39	32,064.35	31,660.17	[23,409, 42,038]	
NRSE	31	6157.46	6990.68	[4089, 8813]	
LOS					
Etiology					
New‐onset, remote	21	10.33	10.22	[6.50, 15.01]	**0.006**
Acute symptomatic	46	18.41	13.63	[14.50, 22.51]	
Remote	69	10.39	11.17	[8.00, 13.20]	
other	16	15.69	17.14	[8.80, 23.74]	
Severity					
RSE	34	11.50	9.36	[8.60, 14.70]	**< 0.001**
SRSE	43	21.40	15.86	[16.90, 26.54]	
NRSE	33	10.85	11.38	[7.30, 14.91]	

*Note:* Number of patients with missing severity classification among those with available cost data: *n* = 40/142; among those with available LOS data: *n* = 42/152. Values in bold indicate *p* < 0.005.

Abbreviations: CI, confidence interval; LOS, length of inpatient stay; NRSE, non‐refractory status epilepticus; RSE, refractory status epilepticus; SD, standard deviation; SRSE, super‐refractory status epilepticus.

To identify independent cost drivers, a multivariable GLM with Gamma distribution and log link was performed (Table 4, *n* = 92 with complete data). After adjustment for all covariates, mechanical ventilation was the strongest statistically significant independent predictor of inpatient costs (Wald *χ* ^2^ = 16.975, *p* < 0.001), with ventilated patients incurring 2.9‐fold higher costs compared to non‐ventilated patients (Exp(*B*) = 2.918, 95% CI 1.753–4.857; adjusted mean €19,189 vs. €6576). Etiology was overall significant (Wald *χ* ^2^ = 7.650, *p* = 0.022), with acute symptomatic SE associated with the highest adjusted mean costs (€16,005) compared to remote SE (€8754) and new‐onset remote SE (€10,117). SE severity, age and pre‐admission mRS did not reach statistical significance after multivariable adjustment (all *p* ≥ 0.084).

**TABLE 4:** Multivariable generalized linear model (GLM) for inpatient costs.

Variable	B	Exp(*B*) (95% CI)	Wald *χ* ^2^	*p*
Mechanical ventilation				
Yes	1.071	2.918 (1.753–4.857)	16.975	**< 0.001**
SE severity				
NRSE	−0.040	0.961 (0.585–1.580)	0.024	0.876
Etiology^a^				
Acute symptomatic	0.459	1.582 (0.874–2.865)	2.291	0.130
Remote	−0.145	0.865 (0.456–1.640)	0.197	0.657
Continuous covariates				
Age (per year)	0.010	1.011 (0.999–1.023)	2.986	0.084
mRS before admission (per point)	0.090	1.094 (0.988–1.211)	2.981	0.084

*Note: n* = 92 patients with complete data. Reference categories: mechanical ventilation = no; SE severity = RSE/SRSE; etiology = new‐onset remote. Value in bold indicate *p* < 0.005.

Abbreviations: B, regression coefficient; CI, confidence interval; Exp(*B*), cost multiplier relative to reference; mRS, modified Rankin Scale; NRSE, non‐refractory SE; RSE, refractory SE; SRSE, super‐refractory SE.

^a^ Overall Wald *χ* ^2^ = 7.650, df = 2, *p* = 0.022.

### 3.4 Ventilation

Less than a third of all patients required ventilation (29%). The mean duration of ventilation was 311.3 ± 495.7 h (median = 121, range = 1–2760), and 75% of all ventilated patients were ventilated for at least 24 h.

Ventilation (*n* = 153) was significantly associated with a longer LOS (*p* < 0.001), discharge destination (no discharge to home, *p* < 0.001), remote (*p* = 0.002) or new‐onset remote (*p* = 0.005) SE, and a higher mRS at admission (*p* = 0.022). However, it was not associated with age (*p* = 0.830), sex (*p* = 0.992), greater disability at discharge (*p* = 0.514), and follow‐up (*p* = 0.160). A longer duration of ventilation was significantly associated with greater disability (higher mRS) at discharge (*p* = 0.010).

### 3.5 Discharge Destination

Figure 3 shows the distribution of mortality and discharge destination by SE severity. The discharge destination (*n* = 105) was associated with the clinical course of SE. Most patients with NRSE were discharged home (*p* = 0.028). Discharges to home were least common among the patients with acute symptomatic SE (29%, *p* = 0.692), and most common among patients with remote SE (63%, *p* = 0.663; new‐onset remote *p* = 0.724). In‐hospital mortality was greatest among those with acute symptomatic SE (21%), followed by those with SRSE (11%), new‐onset remote SE (9%), remote SE (4%), and NRSE (2%).

**FIGURE 3:**
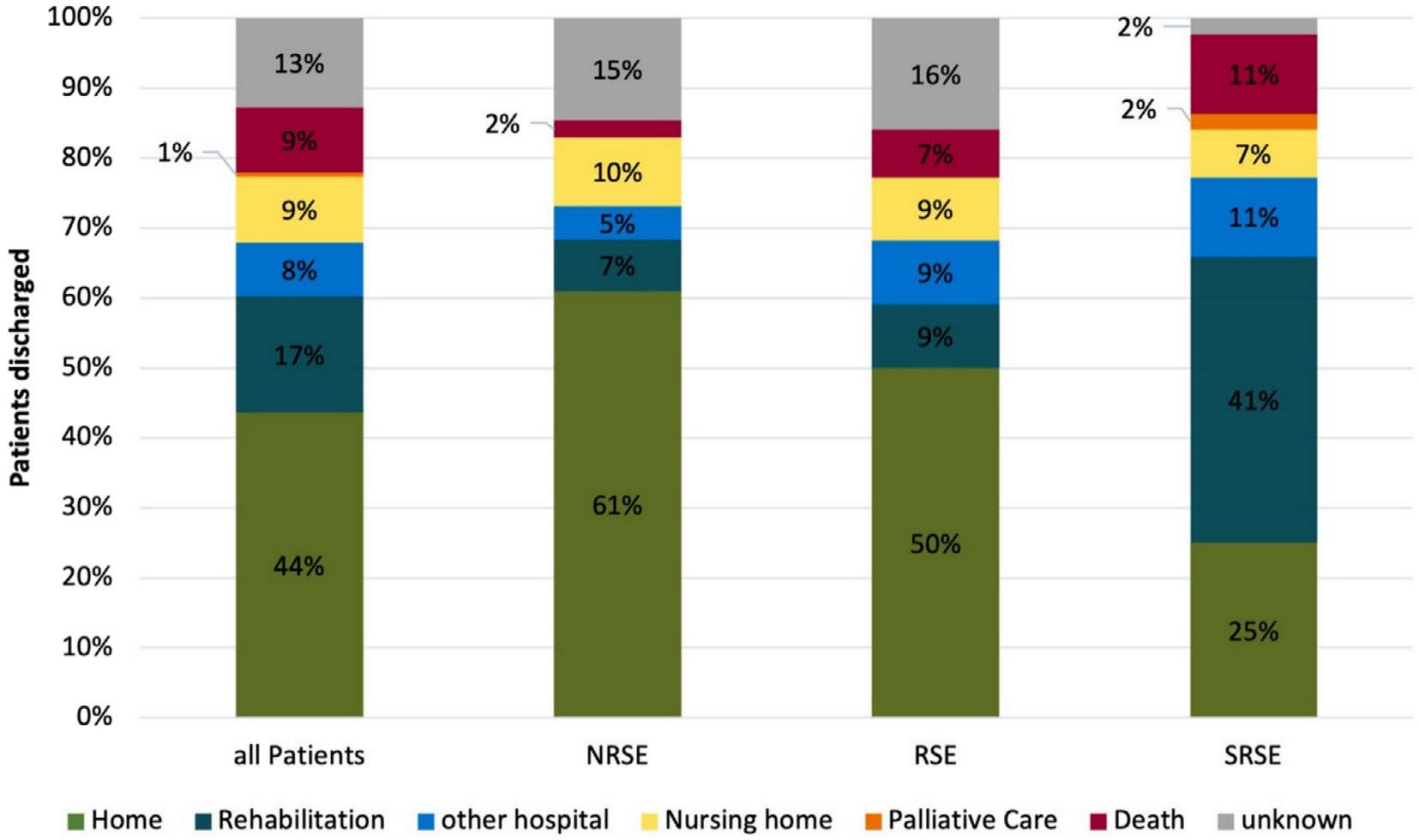
Mortality and discharge destination rates of all patients with SE by SE course. NRSE, non‐refractory status epilepticus; RSE, refractory status epilepticus; SRSE, super‐refractory status epilepticus.

### 3.6 30‐Day Follow‐Up

At 30‐day follow‐up, quality of life and mood were assessed in a subset of patients (*n* = 56–58, Table 5). On the EQ‐5D‐5L, patients reported most impairment in usual activities (mean 1.98 ± 0.90), mobility (1.95 ± 0.87) and self‐care (1.95 ± 0.89), with less impairment in pain/discomfort and anxiety/depression (both 1.48). The EQ‐VAS indicated a mean self‐rated health of 51.12 ± 23.75 (range 10–100) and the NDDI‐E revealed possible depressive symptoms (score ≥ 16) in 32.1% of assessed patients (mean score 11.64 ± 5.29). Functional independence, as assessed by the Barthel Index, showed a mean score of 55.45 ± 40.69 (*n* = 55), indicating moderate dependency on average, with 32.7% achieving full independence (score = 100). The mean mRS score at follow‐up was 2.95.

**TABLE 5:** Outcome at 30‐day follow‐up.

	*n*	Mean	SD
EQ‐5D‐5L			
Mobility	58	1.95	0.87
Self‐care	58	1.95	0.89
Usual activities	57	1.98	0.90
Pain/discomfort	58	1.48	0.54
Anxiety/depression	58	1.48	0.63
Health compared to last year	58	2.24	0.73
EQ‐VAS			
Visual Analogue Scale (0–100)	58	51.12	23.75
NDDI‐E			
NDDI‐E total score	56	11.64	5.29
NDDI‐E binary	56	1.32	0.47
Barthel Index			
Barthel Index (0–100)	55	55.45	40.69
mRS at 30‐day follow‐up			
mRS (0–5)	59	2.95	1.9

*Note:* NDDI‐E positive screen: 1 = score < 16 (no depression), 2 = score ≥ 16 (possible depression); EQ‐5D‐5L dimensions scored 1–3 (1 = no problems, 2 = some problems, 3 = extreme problems/unable); EQ‐VAS: 0–100 (0 = worst, 100 = best health).

Abbreviations: mRS, modified Rankin Scale; SD, standard deviation.

## 4 Discussion

This prospective study, to our knowledge, provides the first population‐based data on costs following the revised definition of SE. It found that the mean cost was €10,543 per patient, and the mean LOS was 11 days, giving a mean cost of €939 per day of treatment. The incidence of SE based on the new definition, determined as part of this survey, was 29.5 per 100,000. Therefore, it is estimated to affect approximately 24,600 patients annually, resulting in annual costs of around €259 million in Germany. While the costs per patient have decreased, the annual costs remain comparable to those in a previous study (more than €200 million) [6] due to the increase in incidence. Data from an Austrian study show that the incidence of SE did not change significantly even during the Covid pandemic; the findings therefore also apply to this period [23].

A previous study published in 2016 examined the costs of treating SE at three university hospitals in the middle of Germany between 2013 and 2014, based on the previous incidence. They estimated costs of €14,946 per admission and €787 per treatment day, with a mean LOS of 19 days [6]. A 2014 review by Kortland et al. estimated that the mean cost per patient was €13,774 (at the current exchange rate) and the mean LOS was 13 days [24], which is similar to the mean LOS reported in the USA in 2008 (12.9 days) [25]. Therefore, the mean cost per patient and LOS in our cohort were lower than those reported in earlier studies [6, 24].

A study examining recent data in a cost‐of‐illness review for SE in Europe reported comparable outcomes for adult patients, with daily costs ranging from €545 to €787 and significantly longer LOS (mean = 19.0–21.2 days) [26]. One possible explanation may be earlier recognition and treatment of SE following the revised operational definition. However, differences in case mix, study settings, and participating centres between studies may also contribute to these differences. The 2016 study was conducted in three tertiary epilepsy centres, whereas our study included a broader network of 14 hospitals and therefore reflects a more heterogeneous patient population [6]. The etiology of SE is also an important consideration, as it plays a significant role in the disease course. Cerebrovascular causes were the most common among the patients included in our study. Nonetheless, advances in treatments for these diseases, particularly lysis and thrombectomy, as well as improved imaging, morphological recognition, and faster diagnosis, all contribute significantly to a reduction in mortality, as well as better functional outcomes and less severe disease courses [27].

As DRG‐based reimbursement has been shown to influence hospital length of stay and resource utilization in epilepsy care, differences in observed costs may partly reflect reimbursement‐related factors rather than clinical severity alone [28]. Indeed, more than half of the recorded SE durations were between 5 and 30 min. Additionally, healthcare spending in Germany has increased steadily, with a 4.9% increase recorded in 2019 compared to 2018 [29]. Owing to the limited follow‐up period of 30 days inherent to the prospective study design, conclusions regarding healthcare costs and patterns of healthcare utilization before and after a status epilepticus event are necessarily restricted. In contrast, analyses based on longitudinal health insurance claims data have demonstrated a clear association between SE and increased utilization of both inpatient and outpatient healthcare services in the subsequent year, with hospital‐related expenditures constituting the predominant cost component [30].

Acute symptomatic SE was associated with the highest unadjusted costs and the longest LOS, comparable to previous studies [6]. An important reason for this is that SRSE occurred more frequently in these cases than in the other etiologies.

SRSE was also associated with the highest unadjusted costs and longer LOS. A cross‐sectional study in the USA among 43,988 patients also showed that those diagnosed with SE in a premier healthcare database had costs and LOS that increased with severity (RSE) and duration [31]. Compared with earlier studies, lower costs and shorter LOS were observed for patients with SRSE in our cohort. However, these differences should be interpreted with caution and may reflect variations in case mix, treatment settings, and changes in clinical practice over time [6]. The higher costs and longer LOS associated with SRSE can be attributed to the greater disability (higher mRS), higher STESS, and longer duration of ventilation on average (363 h vs. 7 h for RSE) of these patients. Determining the degree of SE refractoriness requires detailed documentation of treatment sequences and responses to anti‐seizure medications. In this multicenter study, such information was not always consistently available in routine clinical documentation during acute emergency care, which explains the proportion of cases that could not be classified.

This study has several considerations. It excluded children and adolescents (aged 0–17 years) [32], as well as patients with acute hypoxic brain damage. Therefore, no statement can be made regarding whether their inclusion would alter the incidence and factors driving costs. An international comparison is also challenging because not all countries categorize costs in a similar manner to the G‐DRG [33], and the definition of SE is not uniform; for example, the G‐DRG advocates treating focal and generalized SE after 5 min [34], whereas the ILAE defines generalized SE after 5 min, but focal SE after 10 min of seizure activity [9]. Although extensive efforts were undertaken to maximize case ascertainment, including prospective reporting from all participating hospitals and retrospective database screening at the major university centres, no formal capture‐recapture analysis or external validation of case completeness was performed. Therefore, some degree of underascertainment cannot be completely excluded. Furthermore, a significant proportion of patients could not be classified, for example, in terms of SE severity or mRS score at discharge, primarily due to incomplete documentation in acute emergency care and early discharges. Although the analyses were conducted based on the available cases, this may have led to a bias. Loss of follow‐up could primarily affect patients with severe cases. If the unclassified patients include a disproportionately high number of milder or more severe SE cases, the estimates of costs and length of stay for the severity subgroups may be too low or too high. These limitations are inherent in prospective real‐world studies conducted in various emergency settings and should be considered when interpreting the cost analyses.

Tracing costs back to the disease that causes the costs is often complex, especially due to common cerebrovascular causes. The costs associated with SE were lower than for other common neurological conditions, such as stroke. Direct costs from stroke in Germany ranged from €14,240 to €44,060 per patient [35]. In addition, SE cannot be coded and reimbursed as precisely as stroke, posing challenges for cost studies seeking to examine the causes of economic differences or cost trends. While SE has its own International Classification of Diseases, Tenth Revision (ICD‐10) code (G41), this code has not been adapted to the new ILAE classification system, which is also used in Germany and does not reflect the course, etiology, or severity. Whether an SE event is NRSE, RSE, or SRSE is clearly reflected in the costs, as our results have demonstrated. However, there is no way to differentiate between these forms of SE in the ICD‐10 coding system, and therefore, there is no way to reimburse and track them accordingly [36]. In multivariable analysis, mechanical ventilation emerged as the strongest independent predictor of inpatient costs, highlighting the major impact of intensive care resource utilization. Although higher SE severity was associated with increased costs in univariable analyses, this association was no longer significant after adjustment for clinical covariates, suggesting that the need for ventilatory support may be a more important determinant of inpatient costs.

Willems et al. compared the impact of the introduction of the G‐DRG system in 2003 on LOS for patients with epilepsy. The mean LOS was shorter during the period 2000–2020, with a turning point in 2006. The proportion of patients with a LOS of 1–7 days increased by 17.5% after its introduction, while the proportion of patients with a LOS of 8–14 days decreased by 3.7%. However, it could not be determined with certainty whether the health care services also improved [28]. For SE, there are no data on the impact of the G‐DRG system. Whether the lower costs and reduced LOS are simply an effect of the change in definition or due to the introduction of a new classification system requires further investigation. Although this approach reflects the actual billing practices of hospitals, DRG‐based estimates may not fully reflect the actual resource utilization associated with individual patients.

In half of all patients in whom the duration of SE was recorded, the SE lasted less than 30 min. The mean duration was approximately 8.3 h, significantly shorter than the 18.6 h reported in 2001 [1]. However, a shorter mean duration was to be expected with the new definition due to earlier treatment, which raises the question of whether SE was initially treated according to guidelines. Prehospital care plays an important role, where patients experiencing SE receive initial treatment. In 67.2% of the patients in our study, benzodiazepine (47.6%) or anti‐seizure medication (19.6%) was not administered at a guideline‐compliant dose. Moreover, in 22.3%, an anti‐seizure medication was administered directly before benzodiazepines. In the SENSE study and other cohort studies, which investigated this challenge in treatment, the mean drug doses were not guideline‐compliant: the mean intravenous bolus was 0.029 mg/kg for lorazepam, 0.071 mg/kg for midazolam, and 20 mg/kg for levetiracetam [34, 37]. Further studies on preclinical care are crucial for identifying inconsistencies in acute seizure treatment, while experts tend to recommend even earlier treatment and guideline enforcement should be adjusted accordingly [3].

The results of the 30‐day follow‐up suggest that SE is associated, at the time of the follow‐up assessment, with significant residual functional impairment and a considerable burden of depressive symptoms, which underscores the need for a structured neuropsychological assessment and psychological support following SE.

Our findings are consistent with previous studies indicating that refractory and super‐refractory status epilepticus are major drivers of healthcare costs and resource utilization [6, 31, 36]. By providing population‐based estimates from a mixed network of hospitals, our study adds to the international literature on the economic burden of status epilepticus.

## 5 Conclusion

Mechanical ventilation was identified as the strongest independent predictor of higher inpatient costs, with etiology, particularly acute symptomatic SE, also significantly associated with cost variation. Super‐refractory SE and acute symptomatic etiology were associated with prolonged hospitalization and adverse outcomes.

Early recognition of these high‐risk constellations may support clinical decision‐making and help optimize monitoring, treatment escalation, and allocation of intensive care resources. These findings provide a basis for future cost‐effectiveness studies in status epilepticus in the context of increasing healthcare demands.

## Author Contributions

**Clara Jünemann:** writing – original draft, data curation, formal analysis. **Meike Menche:** writing – review and editing. **Jens‐Peter Reese:** writing – review and editing. **Felix Rosenow:** writing – review and editing. **Martin Jünemann:** writing – review and editing. **Christoph Best:** writing – review and editing. **Michael Teepker:** writing – review and editing. **Christian Roth:** writing – review and editing. **Ingrid Sünkeler:** writing – review and editing. **Elisabeth Pryss:** writing – review and editing. **Leona Möller:** writing – review and editing. **Katja Menzler:** writing – review and editing. **Lars Timmermann:** writing – review and editing. **Susanne Knake:** writing – review and editing. **Adam Strzelczyk:** conceptualization, supervision, writing – review and editing.

## Conflicts of Interest

C. Jünemann: received fellowships from ILAE, DGFE, and DGN. K. Menzler received speaker honoraria and consultant fees from Bial, UCB and Eisai. L. Timmermann: received occasional payments as a consultant for Boston Scientific, L.T. received honoraria as a speaker on symposia sponsored by Boston Scientific, AbbVIE, Novartis, Neuraxpharm, Teva, the Movement Disorders Society und DIAPLAN. The institution of L.T., not L.T. personally received funding by Boston Scientific, the German Research Foundation, the German Ministry of Education and Research, the Otto‐Loewi‐Foundation and the Deutsche Parkinson Vereinigung. Neither L.T. nor any member of his family holds stocks, stock options, patents or financial interests in any of the above mentioned companies or their competitors. L. Timmermann serves as the president of the German Neurological Society without any payment or any income. S. Knake received speaker honoraria from Arvelle Pharma, Bial, Eisai, Epilog, Desitin, Kanso, Merck Serono, UCB Pharma and Zogenix. A. Strzelczyk reports personal fees and grants from Angelini Pharma, Biocodex, Desitin Arzneimittel, Eisai, Jazz Pharmaceuticals, Longboard, Lundbeck, Neuraxpharm, Stoke, Takeda, UCB Pharma, and UNEEG Medical. The other author declares no conflicts of interest.

## Data Availability

Research data are not shared.
